# Refractory ulcerations treated with timolol due to bullous pemphigoid in the setting of chronic cutaneous graft-versus-host disease

**DOI:** 10.1016/j.jdcr.2025.09.016

**Published:** 2025-09-25

**Authors:** Madelyn Kumar, Farrah L. Ezzeddine, Lauren Guggina

**Affiliations:** a Department of Dermatology, Brigham and Women’s Hospital, Boston, Massachusetts; b Dana Farber Cancer Institute, Center for Cutaneous Oncology, Boston, Massachusetts

**Keywords:** bullous pemphigoid, graft-versus-host disease, hematopoietic stem cell transplant, timolol, ulcerations

## Introduction

Chronic graft-versus-host disease (GVHD) is a common complication of allogeneic hematopoietic stem cell transplantation (HSCT), affecting 30% to 70% of patients.^1^ Sclerotic features are a diagnostic feature of chronic GVHD and may be associated with poor wound healing, impaired lymphatic drainage, and ulcers.^1^ Bullous pemphigoid occurring in the context of GVHD has been infrequently reported in the literature, with diagnosis typically based on clinical presentation and histopathological findings. Additionally, autoimmune bullous eruptions are debatably a rare manifestation of GVHD.^2^, ^3^, ^4^, ^5^ Timolol has previously been shown to help with healing of refractory ulcerations.^6^ Herein, we present a case of bullous eruption and ulcerations in a patient with GVHD following HSCT, which ultimately resolved with the therapeutic addition of timolol. Our goal is to contribute to the understanding of this rare manifestation and share our diagnostic and therapeutic approach.

## Case report

**Fig 1:**
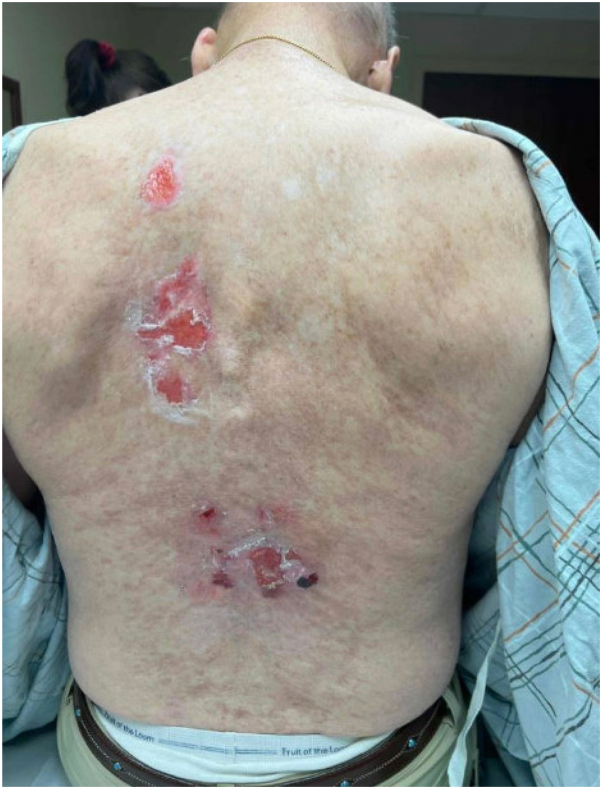
Shallow ulcerations on the lower back at initial presentation, with a background of sclerotic chronic GVHD. *GVHD*, Graft-versus-host disease.

A 67-year-old male presented with 3 months of painful ulcerations on the back (Fig 1). He had previously used topical lidocaine for symptomatic relief. His history was notable for a matched unrelated donor allogeneic bone marrow transplant performed 24 years prior to presentation, complicated by sclerotic cutaneous GVHD of the trunk that was stable and asymptomatic. He discontinued systemic immunosuppressive therapy for GVHD within the same year of the transplant. He reported no systemic or additional cutaneous symptoms.

**Fig 2:**
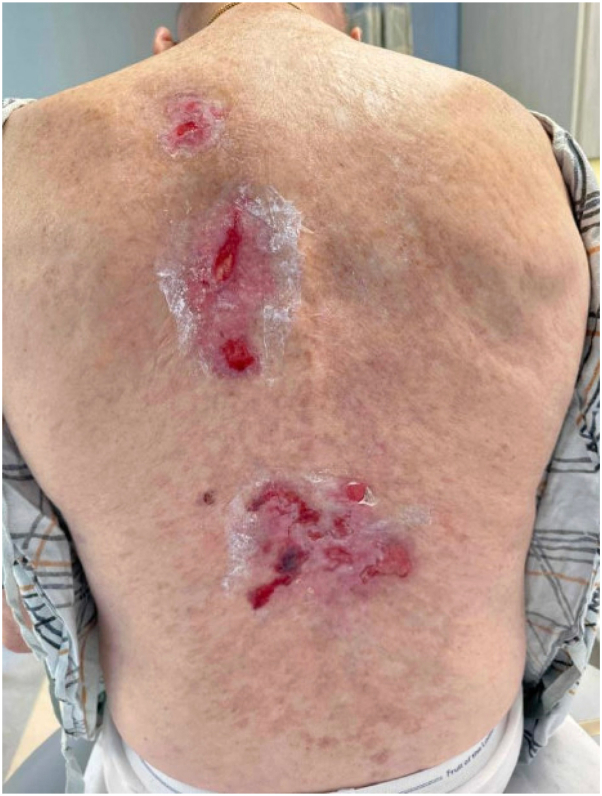
Hemorrhagic bullae overlying areas of sclerosis on the lower back, noted during clinical progression.

**Fig 3:**
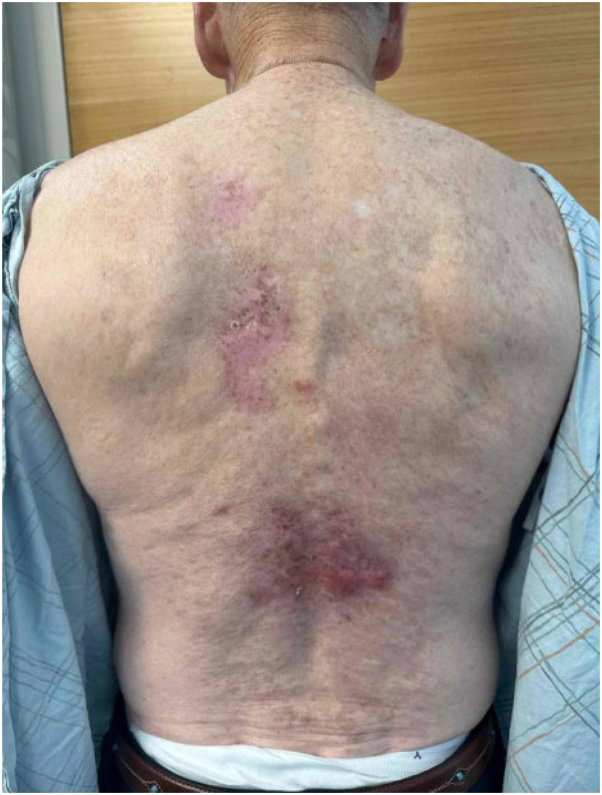
Resolution of erosions and ulcers following treatment with topical timolol and clobetasol.

Examination of the back revealed multiple 2 to 3 cm shallow ulcerations and hemorrhagic bullae with a background of ongoing sclerotic GVHD, with reticular hyperpigmented patches with underlying sclerosis on the abdomen, flanks, axilla, and lower back. Two punch biopsies were obtained, for hematoxylin and eosin and direct immunofluorescence (DIF), and demonstrated epidermal erosion with overlying neutrophilic parakeratosis and superficial to mid-dermal florid mixed inflammatory infiltrate with lymphocytes, neutrophils, and eosinophils on the lower back. DIF showed granular and linear fibrin, immunoglobulin G, C3, and focal immunoglobulin M deposition along the basement membrane. Based on these findings, differential diagnosis from dermatopathology included GVHD, infection, and immunobullous disorder (eg, bullous pemphigoid). Wound culture revealed *Stenotrophomonas maltophila* and he initiated wound care with daily chlorhexidine gluconate solution (Hibiclens), medical honey-base dressings (Medihoney) to open erosions, and clobetasol 0.05% to the edge of ulcerations and active inflammatory plaques, followed by Allevyn foam dressings. The patient demonstrated no improvement at a 9-week follow-up (Fig 2). Indirect immunofluorescence confirmed suspected bullous pemphigoid and timolol 0.5% ophthalmic solution was added to his wound care regimen and applied 1 drop per cm^2^ twice daily, with approximately 2 months of use. Subsequent resolution of his ulcerations was noted at follow-up 8 weeks later (Fig 3). He continues to be asymptomatic without current active therapy.

## Discussion

We present a case of ulcerations and bullae caused by bullous pemphigoid occurring in a post-HSCT patient with sclerotic-type chronic GVHD. We propose that this case of bullous pemphigoid was a manifestation of active GVHD in our patient. Histopathologic findings in cutaneous chronic GVHD typically include interface dermatitis with keratinocyte necrosis, lymphocyte satellitosis, and vacuolar changes.^7^ In this patient, biopsy findings were consistent with cutaneous GVHD; however, his clinical presentation with erosions, bullae, and the presence of eosinophils and prominent dermal inflammation as well as a positive DIF raised suspicion for bullous pemphigoid. The sudden appearance of bullae and DIF revealing linear deposition along the basement membrane confirmed the diagnosis. Bullae are rare manifestations of severe acute GVHD^2^ and are even more rarely associated with chronic GVHD, and we present this case to show that 1 must consider the possibility of autoimmune bullous diseases arising as a complication of GVHD.

Additionally, we present this case as it is the first case displaying the effectiveness of timolol for nonhealing ulcerations in the setting of bullous pemphigoid or GVHD. Potential mechanisms by which beta-blockers may influence wound healing include promoting keratinocyte and fibroblast migration, reducing inflammation, and enhancing angiogenesis.^8^

Two previous case reports described post-HSCT bullous pemphigoid diagnosed via positive immunofluorescence, hematoxylin and eosin staining, and clinical findings.^4^^,^^5^ Treatments included minocycline and nicotinamide in combination with prednisolone and cyclosporine,^4^ and dual CD20 and CD25 chimeric monoclonal antibodies.^5^ Immunosuppressive agents, including topical and systemic corticosteroids, remain the cornerstone of treatment for both bullous pemphigoid and GVHD.^1^^,^^9^ Timolol, an ophthalmic beta-blocker, has shown off-label efficacy in chronic ulcers.^6^ However, its mechanism of action remains unclear. In our case, clobetasol and timolol were effective in resolving our patient’s ulcerations.

It is important to note that systemic absorption can occur with topical application of timolol and its systemic side effects are those typical of beta-blockers, including bradycardia, hypotension, bronchospasm, and central nervous system effects. The standard ophthalmic dosing—one drop of 0.5% solution twice daily—has been associated with systemic adverse effects,^10^ and application of similar doses per cm^2^ of ulcerated skin may exceed this threshold. Clinicians should exercise caution when prescribing timolol in this setting, especially in patients with underlying cardiovascular or pulmonary conditions.

## Conflicts of interest

None disclosed.
